# Route of Delivery in a Patient with Vaginal Stenosis from Stevens-Johnson Syndrome and Review of the Management of Genital Complications

**DOI:** 10.1089/whr.2024.0074

**Published:** 2024-09-06

**Authors:** Meng-Chen Tsai, Geng-Hao Bai, Heng-Kien Au

**Affiliations:** ^1^ Department of Obstetrics and Gynecology, Taipei Medical University Hospital, Taipei, Taiwan.; ^2^ Department of Internal Medicine, National Taiwan University Hospital, College of Medicine, National Taiwan University, Taipei, Taiwan.; ^3^ Department of Obstetrics and Gynecology, School of Medicine, College of Medicine, Taipei Medical University, Taipei, Taiwan.

**Keywords:** cesarean section, genital management, Stevens-Johnson syndrome, vaginal stenosis

## Abstract

**Background:**

Stevens-Johnson syndrome (SJS) and toxic epidermal necrolysis (TEN) are severe dermatological conditions, predominantly affecting women with mortality rates of 4.8–48%. Antibiotics are common triggers. They cause painful mucous membrane erosions in various body parts. Treatment involves steroids, creams, and therapy. Pregnant women with SJS-related vaginal stenosis face challenges of delivery route.

**Case Report:**

A 34-year-old primigravida woman presented at term with vaginal stenosis consequent to a 10-year-history of Stevens-Johnson syndrome triggered by cephalosporin. On pediatric Pederson speculum examination, vaginal stenosis, adhesion, scarred cervix, telangiectasis of the vaginal mucosa, and moderate bleeding after examination were noted. The risks of severe genital tract laceration and excessive bleeding from vaginal birth was discussed with the couple. Shared clinical decision making was reached to undergo a cesarean delivery.

**Conclusion:**

SJS and TEN can result in severe genital complications in women, sometimes requiring cesarean sections due to genital scarring.

## Introduction

Stevens-Johnson syndrome (SJS) and toxic epidermal necrolysis (TEN) are urgent dermatological conditions characterized by extensive epidermal necrosis and sloughing. Moreover, females are more frequently affected than males, with a gender ratio of approximately 1.5:1. Mortality rates stand at 4.8–9% for SJS, 19.4–29% for SJS/TEN, and 14.8–48% for TEN.^1^ While various factors such as infections and underlying malignancies have been linked to SJS, drugs remain the primary triggering agents. Commonly implicated drugs encompass sulfa derivatives, nonsteroidal anti-inflammatory drugs, penicillin-related and cephalosporin antibiotics, antiepileptics, allopurinol, and terbinafine. Painful mucous membrane erosions are prevalent, potentially affecting multiple sites including the lips, oral cavity, conjunctiva, nasal cavity, urethra, vagina, and gastrointestinal tract during the disease course.^2^ In SJS, vulvovaginal involvement has been reported in 12.7% of women,^3^ whereas in TEN, acute vulvovaginal involvement can reach up to 70%, with up to 28% of female patients developing chronic vulvovaginal complications.^4^ Treatment involves the use of potent topical steroids, nonirritating barrier creams, vaginal estrogen, menstrual suppression, and pelvic floor physical therapy.^3,4^ Nevertheless, there are currently no established guidelines for the early detection and prevention of vaginal stenosis sequelae in child-bearing age females affected by SJS/TEN nor are there recommendations regarding the optimal delivery method for pregnant women experiencing vaginal stenosis resulting from SJS.

In this report, we present a case of a pregnant woman at full term who developed vaginal stenosis as a consequence of prior Stevens-Johnson syndrome induced by cephalosporin antibiotics. Due to the substantial risks associated with severe vaginal laceration and excessive bleeding, we decided to perform a cesarean delivery, which was carried out successfully.

## Case Presentation

A 34-year-old primigravida at 39 4/7 weeks of gestation presented with vaginal bleeding. She had a 10-year history of Stevens-Johnson syndrome induced by NSAIDs and Cefaclor treatment. She was admitted at another hospital because of corneal, oropharyngeal, and vulvar ulcerations. A dermatologist had suggested a skin biopsy which the patient refused. Based on the sudden onset of systemic mucocutaneous eruptions shortly after a history of drug exposure, a diagnosis of SJS was made. The patient was not sexually active, and thus a gynecologist consultation was not made. Her symptoms were gradually improved with steroid treatment. She was discharged 21 days after admission. Since then, she avoided using the triggering medications, and she did not experience SJS again. The patient was sexually active after getting married 4 years before admission. Three months before conception, she has had a transvaginal ultrasound examination for menstrual irregularity. She had first prenatal visit at our hospital at 12 weeks of gestation. She reported a natural conception. She did not mention having a difficult vaginal sex. She was referred to have a consultation by a rheumatologist who advised to do an adverse drug reaction testing, though which was not completed before labor onset. At 35 weeks of gestation, she was advised to massage perineum daily. On routine antenatal group B streptococcus (GBS) screening, the patient refused the introduction of vaginal swab but accepted the anal swab. After admission for labor, she expressed worsening vaginal pain on digital cervix examinations. Only then she disclosed that she often experienced vaginal discomfort and bleeding during vaginal sex with deep penetration. However, she did not seek medical assistance seeing that the couple was the first and only sexual partner to each other. On pediatric Pederson speculum examination, vaginal stenosis, and cervical-vaginal adhesions, stenotic and scarred cervix, and telangiectasis of vaginal mucosa were noted. Transabdominal sonography confirmed vertex presentation, and a non-stress test showed a healthy fetal heart rate of 130 beats/min. The increased risks of severe genital tract laceration and excessive bleeding from vaginal birth, and risks and benefits of cesarean birth versus vaginal birth was discussed with the couple. After extensive counseling and their questions were answered, shared clinical decision making was reached to undergo a cesarean delivery. A live female infant weighing 2,282 g was delivered with Apgar scores of 9 and 10 at 1 and 5 minutes. Both mother and baby were discharged in good health 48 hours later and remained healthy at the 6-week postnatal review.

## Discussion

Genital manifestations of SJS and TEN encompass erosive and ulcerative vaginitis, vulvar bullae, and vaginal synechiae in females. The most prevalent findings involve vulval erosions and skin sloughing in the mons pubis and perineal region. Moreover, SJS and TEN can lead to complications like sexual difficulties, infertility, or birthing disorders.^5^ A systematic review aimed to characterize risk factors, outcomes, and treatment approaches for SJS and TEN in pregnant women. Common causative medications included antiretroviral therapy (90%), antibiotics (3%), and gestational drugs (2%). Complications comprised preterm labor, vaginal stenosis, and adhesions. The delivery method was unspecified in most case reports, with 20 women undergoing cesarean sections and 22 delivering vaginally.^6^ The literature presents options for managing severe vaginal stenosis in pregnant women, including cesarean section, incision to the obliterated vagina, or vaginal delivery with episiotomy.^7^ In some cases, vaginal delivery was possible even with vaginal stenosis; for instance, El Daief et al. reported a case of a young pregnant woman with a previous history of SJS due to penicillin treatment who had a normal vaginal delivery without complications.^8^ However, Winston and Mastroianni et al. presented a case with a completely obliterated vagina that was not suitable for Cesarean section due to the development of immediate labor and severe orthopnea. A transverse incision was made into the obliterated vagina and a deep right mediolateral episiotomy was made for the emergency delivery.^9^ The case presented by Mousazadeh et al.^10–19^ bore similarities to our case. Both patients had a history of SJS years before pregnancy and opted for a Cesarean section due to vaginal stenosis.

Table 1 summarizes case reports detailing the management of genital complications in pregnant women with SJS and TEN. To prevent vulvovaginal scarring, daily use of a vaginal dilator and early initiation of topical steroids may be necessary. Managing pregnant SJS/TEN patients involves discontinuing causative agents, emphasizing early diagnosis, and monitoring for preterm labor’s impact on both mother and fetus. The delivery mode should align with the severity of genital involvement, necessitating frequent obstetric assessments for vaginal strictures. A multidisciplinary team comprising obstetricians, physicians, anesthetists, neonatologists, and dermatologists is crucial for comprehensive care. In cases where vaginal delivery is planned, cesarean section preparations should also be made.

**Table 1.** Case Reports Literature of SJS/TEN in Pregnancy with Genital Management: Table Adjusted and Completed from Niemeijer et al.^7^ and Sharma et al.^6^

Author/year	Trigger	Disease onset (weeks)	Delivery (weeks)	Genital involvement	Genital management	Delivery (route)	Reference
Winston and Mastroianni, 1954	Penicillin	24–28 (unsure)	33–37	Vagina completely obliterated	Postpartum petrolatum gauze	Vaginal delivery with transverse incision and right mediolateral episiotomy	^9^
Vasicka, 1958	Slight cold	38	40	Eruptions on external genitalia and vagina	Bacitracin ointment and neocortef drops Washed with hydrogen peroxide	Vaginal delivery with midline episiotomy	^11^
Graham Brown, 1981	Pregnancy	33	38	Vulvar ulcers Purulent, blood-stained vaginal discharge	2 % miconazole gel to vulva 0.5% chlorhexidine solution after toilet	Cesarean section (breech presentation)	^12^
Petukhova, 2016	Penicillin/pregnancy	10	38	Horizontal perineal strictures Fusion of the labia majora, minora, and clitoral hood	Vaginal packing and dilators Zinc oxide to erosions Foley until the perineal area fully re-epithelialized Intravaginal Foley 4 months Post-partum, vaginal stent with estrogen cream at night Daily placement of a vaginal dilator with lidocaine jelly	Cesarean section	^13^
Shakoei, 2021	Ondansetron	18	38	Labias and introitus	Clobetasol ointment	Vaginal delivery	^14^
Calley, 2022	Influenza vaccine	31	31 (preeclampsia)	Vulvar erosions	Clobetasol, triamcinolone, and betamethasone ointments	Cesarean section	^15^

The evaluation of pediatric and child-bearing age females affected by SJS and TEN should include the possibility of developing vaginal stenosis, leading to conditions like hematocolpos or vulvovaginal endometriosis. Timely recognition and treatment of vulvovaginal complications are critical to prevent severe long-term consequences. Unfortunately, genital involvement is often not identified until the late stages of the acute SJS/TEN phase, limiting treatment options. In our case, the patient had a 10-year history of SJS with vulvovaginal involvement. Early recognition and intervention could have prevented obstructed labor or subsequent genital tract laceration. Therefore, gynecological examinations of pediatric and child-bearing age females are essential to identify genital adhesions promptly.

Recommendations for managing genital complications during the acute phase are age-stratified. For pediatric patients, a pelvic examination under sedation is recommended for multidisciplinary assessment. If this examination is challenging, the use of topical steroids and estrogen cream, both internally and externally, is advised. In young adults, menstrual suppression is recommended to reduce the risk of vaginal adenosis. Early initiation of intravaginal steroids, estrogen cream, and estrogen ring is essential. Moreover, the use of vaginal dilators or molds is suggested in cases of severe vaginal involvement. Importantly, all patients should receive long-term follow-up care by gynecologists until vaginal erosions are fully healed.^3^ Table 2 additionally provides a summary of the management strategies for established vaginal stenosis in pediatric and child-bearing age individuals. This management entails the separation of adhesions, drainage of hematocolpos, and the placement of vaginal dilators in sexually active patients.

**Table 2.** Case Reports Literature of Vaginal Stenosis Caused by SJS/TEN in Pediatric or Young Adults with Genital Management

Author/year	Trigger	Disease onset (age)	Genital involvement	Symptoms	Genital management	Reference
Hart, 2002	Cephalosporin	11	Vulval and vaginal ulceration	Not yet started to menstruate	Division of adhesions 6 months after *via* vaginoscopy	^16^
Murphy, 1998	Erythromycin	14	Fused vaginal mucosa without opening	5-month history of lower abdominal cramping and amenorrhea	Incision and drainage of hematocolpos	^17^
Marqeutte, 1985	Sulfonamide	21	Partial fusion of labia minor and labia major	Vulvar pain and dyspareunia 1.5 years after	No improvement after megestrol used Excision of the vestibular and perineotomy	^18^
Willson, 1988	Sulfonamide	23	Endometriosis of vulva, vagina, and cervix	Constant vaginal bleeding	Danazol twice daily for 1 year without resolution Midline episiotomy, resection of abnormal epithelium, vaginal gauze, and mold insertion	^19^

In summary, while we have described the case of a pregnant woman who successfully underwent a cesarean delivery despite having developed vaginal stenosis, it underscores the importance of early detection of vulvovaginal involvement in SJS/TEN, appropriate genital management during the acute phase, and careful consideration when managing pregnant women with SJS/TEN.

## Abbreviation Used

SJS: Stevens-Johnson Syndrome
TEN: Toxic Epidermal Necrolysis
